# Tumor and microenvironmental mechanisms of resistance to immunomodulatory drugs in multiple myeloma

**DOI:** 10.3389/fonc.2022.1038329

**Published:** 2022-11-09

**Authors:** Lucia Y. Chen, Sarah Gooding

**Affiliations:** ^1^ Department of Haematology, Oxford University Hospitals NHS Foundation Trust, Oxford, United Kingdom; ^2^ Oxford Centre for Translational Myeloma Research, University of Oxford, Oxford, United Kingdom; ^3^ MRC Molecular Haematology Unit, Weatherall Institute of Molecular Medicine, University of Oxford, Oxford, United Kingdom

**Keywords:** immunomodulatory drugs, cereblon, drug resistance, immune microenvironment, multiple myeloma

## Abstract

Resistance to immunomodulatory drugs (IMiDs^®^) is a major cause of treatment failure, disease relapse and ultimately poorer outcomes in multiple myeloma (MM). In order to optimally deploy IMiDs and their newer derivates CRBN E3 ligase modulators (CELMoDs^®^) into future myeloma therapeutic regimens, it is imperative to understand the mechanisms behind the inevitable emergence of IMiD resistance. IMiDs bind and modulate Cereblon (CRBN), the substrate receptor of the CUL4^CRBN^ E3 ubiquitin ligase, to target novel substrate proteins for ubiquitination and degradation. Most important of these are IKZF1 and IKZF3, key MM survival transcription factors which sustain the expression of myeloma oncogenes IRF4 and MYC. IMiDs directly target MM cell proliferation, but also stimulate T/NK cell activation by their CRBN-mediated effects, and therefore enhance anti-MM immunity. Thus, their benefits in myeloma are directed against tumor and immune microenvironment – and in considering the mechanisms by which IMiD resistance emerges, both these effects must be appraised. CRBN-dependent mechanisms of IMiD resistance, including *CRBN* genetic aberrations, CRBN protein loss and CRBN-substrate binding defects, are beginning to be understood. However, only a proportion of IMiD-resistant cases are related to CRBN and therefore additional mechanisms, which are currently less well described, need to be sought. These include resistance within the immune microenvironment. Here we review the existing evidence on both tumor and immune microenvironment mechanisms of resistance to IMiDs, pose important questions for future study, and consider how knowledge regarding resistance mechanism may be utilized to guide treatment decision making in the clinic.

## Introduction

Immunomodulatory drugs (IMiDs) are a major class of drugs in the treatment of multiple myeloma (MM) that have radically improved patient survival. Both a direct anti-proliferative effect on MM cells and modulation of the immune microenvironment contribute to their efficacy. However, MM eventually relapses during IMiD-based combination therapies due to the emergence of drug resistance. Understanding resistance mechanisms to IMiDs is therefore a vital step toward developing novel therapeutics and improving outcomes for MM patients.

Thalidomide was the first IMiD to show clinical efficacy in MM (1, 2). The first analogue of thalidomide to be approved, lenalidomide, showed superior clinical effectiveness and toxicity profiles in frontline (3), relapse (4) and maintenance settings (5). Pomalidomide, another thalidomide derivative, has shown efficacy in around one-third of lenalidomide-resistant patients and is predominantly utilized in the relapsed-refractory setting (6, 7). Newer derivatives of IMiDs, CRBN E3 ligase modulators (CELMoDs) bind target protein cereblon (CRBN) with greater affinity, leading to faster substrate degradation and therefore more potent anti-proliferative effects. Recent data suggests CELMoDs iberdomide (CC-220) and mezigdomide (CC-92480) may have efficacy in lenalidomide and pomalidomide-resistant patients in ongoing clinical trials (8–10).

## IMiDs act *via* cereblon

CRBN was identified as the primary target of thalidomide in 2010 (11). CRBN is the substrate adaptor of the CRL4^CRBN^ E3 ubiquitin ligase, a cullin-ring ligase composed of damaged DNA-binding protein 1 (DDB1), cullin 4a (CUL4A), and regulator of cullins 1 (ROC1). Subsequent pivotal studies demonstrated that IMiD-bound CRBN recruits novel substrates ikaros (IKZF1) and aiolos (IKZF3) to the CRL4^CRBN^ E3 ubiquitin ligase complex for ubiquitination and subsequent proteosome-mediated degradation (12, 13). A third transcription factor, zinc finger (ZnF) protein 91 (ZFP91), was identified as an IMiD-bound CRBN target (14). Lack or loss of ability to fully degrade ZFP91 may mediate resistance to IMiDs (15), although this has not been reported in myeloma cells (14). The Zn2 and Zn4 domains of ZFP91 and IKZF1/3 respectively share a common motif; it is likely that other ZnF-containing substrates susceptible to IMiD-induced CRL4^CRBN^ binding and degradation exist. The protein specificity of the CRL4^CRBN^ complex is modified by IMiDs with great precision (12). For example, lenalidomide can recruit casein-kinase 1 alpha (Ck1a) in addition to IKZF1 and IKZF3, whereas thalidomide cannot (16).

IKZF1 and IKZF3 are zinc finger transcription factors which regulate normal lymphopoiesis and B-cell development. IKZF1 is required for pro-B cell to pre-B cell differentiation and is essential for VDJ recombination (17). IKZF1 regulates IRF4 expression, critical for plasma cell differentiation *via* the IRF-BLIMP-1 feedback loop. Knockdown of either IKZF1 or interferon regulatory factor 4 (IRF4) results in blockade of plasma cell differentiation (18). IKZF3 is specifically responsible for the differentiation of long-lived high affinity plasma cells in the bone marrow (19).

IMiD-induced CRBN-mediated effects independent of the CRL4^CRBN^ E3 ligase have also been proposed. CRBN was shown to bind and stabilize the CD147-monocarboxylate transporter 1 (MCT1) transmembrane protein (TP) complex in a ubiquitin-independent manner (20), and similarly the amino acid transporter complex LAT1/CD98hc (21). CRBN has a ‘co-chaperone’ function in membrane delivery of these TPs, stabilizing their interaction with the heat shock protein of 90 kDa (HSP90)- ATPase activity 1 (AHA1) chaperone complex. IMiD binding to CRBN, or CRBN loss, abrogates its HSP90 binding so reversing its stabilizing effect on client TPs during maturation. The relative contribution to IMiD efficacy of TP destabilization vs CRL4^CRBN^ E3 ligase-mediated activity remains unclear.

## Effects of IMiD-induced substrate degradation in MM

### Anti-proliferative effect via downregulation of IRF4 and myc proto-oncogene

In physiological B-cell development, MYC is a transcriptional target of IRF4, and the MYC-IRF4 axis is transiently active during active B-cell expansion before MYC is downregulated (22, 23). However, in MM, IRF4 also becomes a transcription target of MYC, resulting in an aberrant autoregulatory network that drives sustained MYC expression and MM cell proliferation. MM cells are ‘addicted’ to both IRF4 and MYC as proliferation-inducing oncogenes, and independent IRF4 or MYC inhibition has been shown to be toxic in MM cell lines (24, 25). In normal B-cell development, IKZF1 negatively regulates MYC. By contrast in MM, due to transcriptional regulation rewiring that is incompletely understood, IMiD-mediated IKZF1 and IKZF3 degradation causes sequential downregulation of MYC and IRF4 proteins, breaking the aberrant oncogenic proliferative drive of the MYC-IRF4 axis to induce cell cycle arrest and eventual apoptosis (26, 27).

### Enhancing anti-tumor immunity

**Figure 1:**
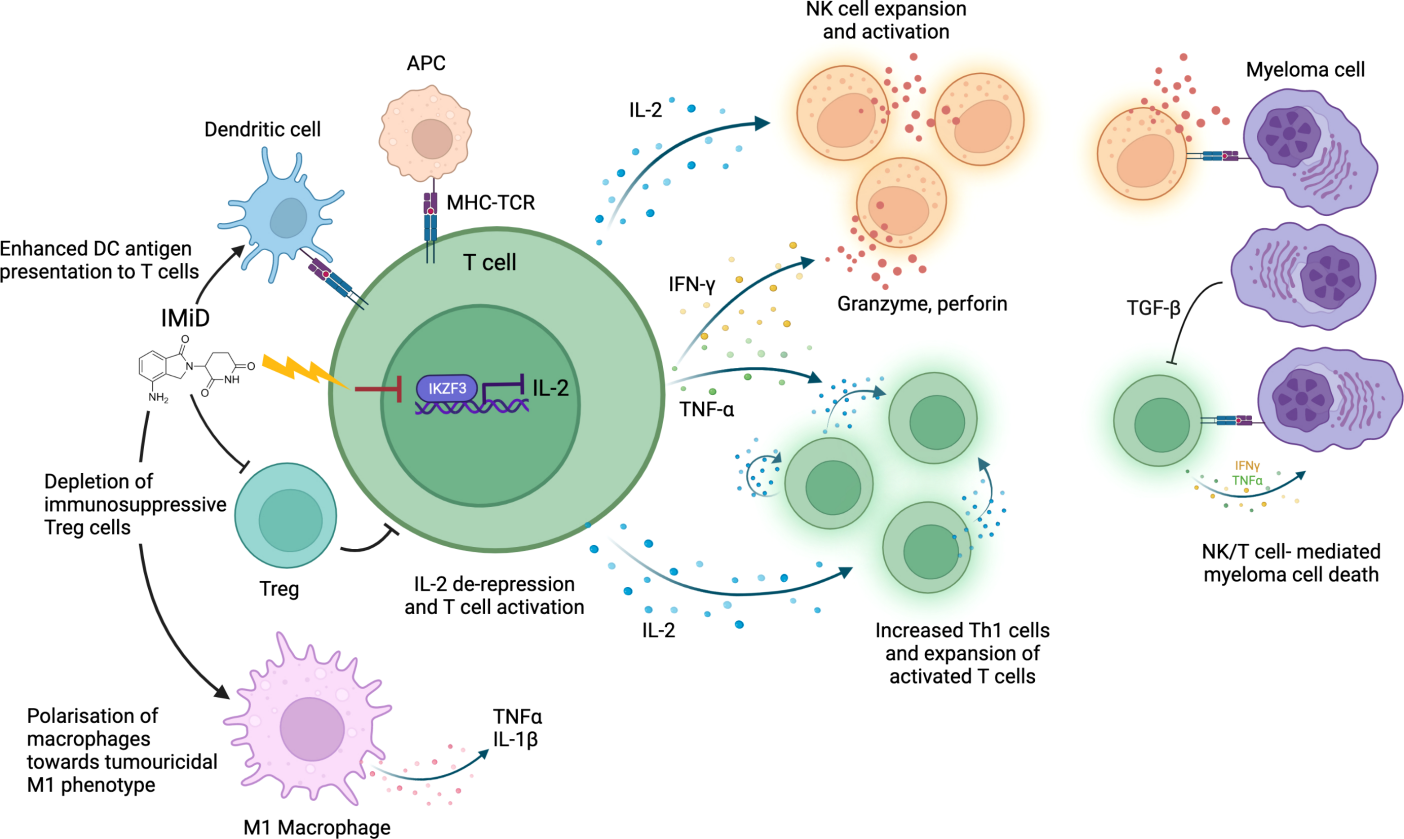
IMiD drug modulation of anti-myeloma immunity. IMiDs de-repress IL-2 transcription via CRBN-mediated IKZF3 degradation. IL-2 production stimulates further cytokine release (including IFNγ and TNFα) leading to T/NK cell expansion and enhanced immune-mediated destruction of myeloma cells. IMiDs further increase anti-myeloma immunity through polarization of macrophages towards an M1 tumoricidal phenotype, stimulation of dendritic cell antigen presentation and depletion of Tregs. Antigen presenting cell (APC); Major histocompatibility complex (MHC); T-cell receptor (TCR).

The immunostimulatory effect of thalidomide on T cells was first discovered several decades ago as part of an effort to understand its anti-inflammatory properties in erythema nodosum leprosum (28). Subsequent studies have demonstrated that IMiDs not only activate T cells but have varying modulatory effects on a range of immune cells, including natural killer (NK) cells, dendritic cells and macrophages (**Figure 1**).

The best studied immunomodulatory effect of IMiDs is the enhancement of T and NK cell-mediated anti-MM cytotoxicity. Immune dysfunction occurs as part of MM pathogenesis: in untreated MM patients an abnormal Th1/Th2 ratio, increased T cell senescence and exhaustion and reduced NK cell levels are seen (29–31). This is partly due to elevated levels of transforming growth factor beta (TGF-β), an immunosuppressive cytokine, secreted by plasma cells and MM microenvironment cells (30). IMiDs help to overcome this T and NK cell dysfunction to restore a degree of anti-MM immune clearance. *In vitro* studies have demonstrated that IMiDs trigger T cell production of cytokines including interferon-γ (IFN-γ) and interleukin-2 (IL-2), which drive T cell clonal expansion (32, 33), and NK cell activation (34). IMiDs also activate CD4^+^ and CD8^+^ T cells by inducing increased dendritic cell (DC) antigen presentation (35), facilitating immune recognition and killing of MM cells. In keeping with *in vitro* studies, T cell profiling studies in IMiD-treated MM patients consistently demonstrate a shift towards activated, proliferative, and cytotoxic T cell profiles (36, 37). Clinical studies also demonstrate an association between strength of T/NK cell activation and degree of clinical anti-myeloma response, implying it is an important mechanism of IMiD efficacy (38, 39). Interestingly, NK cell levels do not correspond to response rates in patients with myelodysplastic syndrome (MDS) on lenalidomide, suggesting that MM cells may be particularly vulnerable to NK cell-mediated cytotoxicity (40).

In addition to promoting T and NK cell-mediated anti-tumor immunity, IMiDs may synergize with immune checkpoint blockade to reduce MM immune tolerance. Reduced Programmed death-1 (PD-1) expression in T and NK cells was seen in MM patient immune cells after lenalidomide treatment (41, 42). Whilst clinical trials of PD-1/PDL-1 inhibitors have been halted in MM following mixed results on safety and efficacy, IMiDs have also been shown to synergize with the widely-used anti-CD38 MM therapies, Daratumumab and Isatuximab. In addition to direct plasma cell toxicity, Daratumumab depletes CD38-positive immunosuppressive regulatory T cells (Tregs) and expands effector CD4 and CD8 T cells as a mode of action (43), but also depletes NK cells. IMiD-daratumumab combinations show enhanced cytotoxic T cell activity and reduced immunosuppressive cells (44). IMiDs may also overcome daratumumab resistance through upregulation of CD38 (45).

The mechanism by which IMiDs activate T and NK cells remains incompletely understood. However, there is evidence that CRBN-mediated IKZF1/3 degradation in immune cells causes much of the immunostimulatory effect. IKZF1 is a known transcriptional repressor of IL-2 and IFN-γ, whilst IKZF3 specifically represses the IL-2 promoter (46, 47); therefore, degradation of IKZF1/3 derepresses IL-2 and IFN-γ leading to T and NK cell activation (48). Studies demonstrate that higher potency IMiDs such as pomalidomide, which bind CRBN with greater affinity, can produce a stronger immunostimulatory effect than less potent IMiDs (38, 49). IKZF1 degradation has also been implicated in polarization of macrophages toward a tumoricidal M1 phenotype by IMIDs (50). CRBN-mediated degradation of a different target, Ck1a, has been shown to modulate interferon pathways (51). However, whether all immunomodulatory effects of IMiDs are CRL4^CRBN^ E3 ubiquitin ligase-dependent remains to be elucidated.

The addition of steroids to IMiDs in MM regimes enhances their anti-proliferative effects but inhibits their immunostimulatory effects. Dexamethasone addition to lenalidomide caused increased MM cell death but blunted IL-2 production by T-cells and reduced NK cell activation (52, 53). Clinical studies in relapsed/refractory MM (RRMM) showed higher response rates on adding dexamethasone to lenalidomide (54), indicating the anti-proliferative effect of IMiDs to be more significant than their immunomodulatory effects. Studies in different disease contexts, such as high-risk smoldering MM (SMM) have demonstrated significant immune cell activation by IMiDs despite steroid use (55).

## IMiD mechanisms of resistance

Resistance emergence to immunomodulatory drugs (IMiDs) is a crucial barrier to prolonging relapse-free survival in MM. Widespread use of IMiDs as maintenance therapy post- autologous stem cell transplant (ASCT) further heightens the need to clarify IMiD resistance mechanisms and the impact of their long-term use on the biology of relapsed MM.

### Cereblon-related IMiD resistance mechanisms

**Figure 2:**
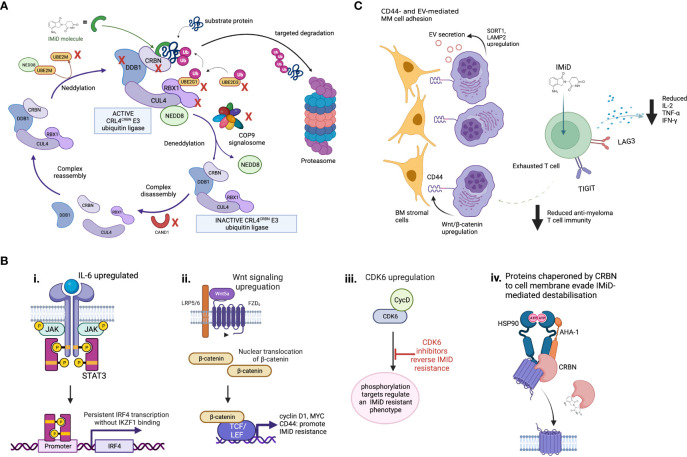
Mechanisms of IMiD drug resistance. **(A)** Proteins involved in maintenance of the CRL4^CRBN^ E3 ubiquitin ligase are required for IMiD drug efficacy. The CRL4^CRBN^ E3 ubiquitin ligase is maintained at an active level by a tightly controlled loop of neddylation and deneddylation, regulators of which include the COP9 signalosome and various E2 ligases. If deneddylation is impaired, CRBN itself may be ubiquitinated in the absense of substrate binding, and targeted for degradation. CRISPR screen evidence shows many of the proteins that regulate the CRL4^CRBN^ E3 ubiquitin ligase are essential for IMiD and novel CRBN-targeting PROTAC agent activity, and their loss may therefore drive drug resistance. Those identified as IMiD-essential in reported screens are marked with a red cross. **(B)** Some proposed CRBN-independent mechanisms of IMiD resistance. (i) Upregulated IL-6/STAT3 signaling drives IKZF1s-independent IRF4 transcription. ii. Upregulated Wnt/β-catenin signaling drives IKZF1/IKZF3 independent MYC transcription, plus CD44. iii. CDK6 overexpression regulates a set of protein targets associated with IMiD resistance. CDK6 inhibition reverses this state. iv. Although mechanism is unknown, in IMiD-resistant states the transmembrane proteins which require CRBN as a chaperone resist IMiD-induced destabilization. **(C)** Potential microenvironmental mechanisms of IMiD resistance. These may include CD44- and extracellular vesicle (EV)-mediated MM cell adhesion to bone marrow (BM) stromal cells, and T cell exhaustion, which is associated with increased checkpoint inhibitor expression, reduced IL2 expression and results in reduced anti-myeloma activity.

Disruption of Cereblon activity is the best understood mechanism of IMiD resistance in MM (**Figure 2**). As CRBN is obligatory for IMiD activity (56), either quantitative or qualitative CRBN defects may contribute to IMiD ineffectiveness or resistance. Quantitative mass spectrometry studies testing the activity and kinetics of CRL4^CRBN^ E3 ubiquitin ligase demonstrate three key factors that could affect its activity levels and cause IMiD resistance: i) levels and stability of E3 ligase components such as CRBN, ii) strength of ligase-substrate interaction induced by the IMiD, and iii) expression level of competing substrates (57). Aberrations to any or all of these factors could cause CRBN-related IMiD resistance.

Levels of CRBN protein or RNA have been demonstrated in several studies to correlate to IMiD response in both MM patients and cell lines (58–60). However, other studies have shown no association between baseline CRBN levels and responsiveness to IMiDs (61–63). The lack of standardized and validated CRBN measurement assays, and the many other factors that contribute to inter-patient variability in CRL4^CRBN^ E3 ubiquitin ligase activity, and indeed IMiD function overall, likely contribute to this discrepancy (62). Over-expression of CRBN has been shown to successfully re-sensitize IMiD-resistant MM cell lines to IMiDs (57, 64), which supports the hypothesis that loss of functional CRBN is a major mechanism of IMiD resistance.

*CRBN* genetic aberrations are the best-validated IMiD resistance mechanism to-date. Although *CRBN* mutations occur infrequently in newly diagnosed (<1%) (65) and IMiD-treated patients (12%) (66), our recent comprehensive whole-genome (WGS) and RNA sequencing analysis revealed a spectrum of genomic and transcriptomic abnormalities beyond single nucleotide variants (SNVs), that may underpin reduced CRBN function in clinical IMiD resistance (67). SNVs, structural variants, exon 10 splicing and gene copy loss were all noted to increase in incidence between newly diagnosed, lenalidomide- and pomalidomide-refractory MM patients, to an incidence of almost one-third in pomalidomide-refractory patients. Such genetic alterations appear to undergo therapeutic selection during IMiD exposure. Lenalidomide-refractory patients with these *CRBN* aberrations were less likely to respond to pomalidomide and had a worse progression-free survival (PFS) irrespective of *CRBN* gene expression levels. Further studies are required to understand the relationship between CRBN genetic disruption, its protein level/function, and differential efficacy between IMIDs.

Genome-wide CRISPR-Cas9 knock out screens in MM have recently highlighted the role of CRL4^CRBN^ E3 ligase associated proteins, including COP9 signalosome (CSN) members and certain E2 ubiquitin-conjugating enzymes, in mediating IMiD sensitivity and resistance through their regulation of CRBN-mediated substrate degradation (68, 69). The CSN complex deactivates the CRL4^CRBN^ E3 ligase through de-neddylation (**Figure 2**), to regulate its activity level. Loss of CSN function therefore increases ligase activity, meaning CRBN may be auto-ubiquitiated and degraded. All CSN subunits are required for its deneddylating activity, and loss of even one subunit can lead in turn to cellular CRBN loss. We have recently used WGS datasets comparing newly diagnosed to lenalidomide- and pomalidomide-refractory MM patients to identify copy loss of chromosome region 2q37, containing CSN members *COPS7B* and *COPS8*, as an increasingly common feature usually occurring exclusively to genetic *CRBN* loss in IMiD-refractory patients. Thus defective CSN regulation of CRBN degradation may be a clinically-relevant mechanism of CRBN-mediated IMiD resistance (70).

Relative affinity of IMiD-induced CRBN-substrate binding contributes to the development of IMiD resistance. CELMoDs have a 20-fold higher binding affinity for CRBN compared to lenalidomide, and consequently result in faster IKZF1/3 degradation and improved efficacy (57). Consequently, CELMoDs can overcome IMiD resistance in MM cell lines (8, 71). Iberdomide has demonstrated a 30% overall response rate in IMiD-exposed patients in phase 1b/2a studies (9). This reversal of IMiD resistance with better kinetics of CRBN-IKZF1/3 binding and degradation suggests that further study of ligase-substrate interactions as resistance mediators is warranted. The ordering of such agents in patient treatment regimes should also be considered, as IMiDs with lower affinity are less likely to have efficacy after resistance to CELMoDs has been acquired. Levels of competitors for substrate binding may also reduce drug efficacy; for example, RUNX1 and RUNX3 transcription factors have been shown to compete with CRBN to bind IKZF1/3, thereby reducing CRBN-IKZF1/3 binding and degradation (72). Inhibition of RUNX proteins in MM cell lines was able to reverse IMiD resistance. In MM cell lines, 244 different CRBN E3 ligase substrates were identified and of these, 46 were able to bind IMiD-bound CRBN, suggesting that they could in theory compete with IKZF1/3 in binding CRBN (73). In one *in vitro* study, overexpression of a substrate known to bind CRBN tightly induced lenalidomide resistance due to reduced degradation of other competing substrates including IKZF1 (57). Other substrates of IMiD-bound CRBN-mediated degradation may contribute to efficacy or resistance; for example, ARID2 is a target of CRBN degradation in pomalidomide-treated patients and may regulate MYC independently of IKZF1/3 (74). Whether ARID2 actively competes with IKZF1/3 for CRBN binding remains unclear. The hypothesis that MM cells could upregulate unrelated CRL4^CRBN^ E3 ligase substrates to compete with IKZF1/3 as a mechanism of resistance to IMiDs requires further interrogation.

Loss of IKZF1 and IKZF3 has also been studied as a potential mechanism for IMiD resistance, but results thus far are inconclusive. In newly diagnosed lenalidomide-treated MM patients, high IKZF1 expression was associated with improved PFS (75, 76). In the pre-ASCT setting, IKZF1/3 protein levels were non-prognostic, and c-MYC was the only downstream CRBN target that impacted survival (76). Studies investigating IKZF1 expression levels in the microenvironment cells of lenalidomide-treated MM patients, suggested a correlation with improved survival (77). However, other studies have demonstrated no significant role for IKZF1/3 levels in predicting sensitivity to IMiDs (61), therefore contentions remain.

### Cereblon-independent IMiD resistance mechanisms

As a significant proportion of IMiD-refractory patients have no detectable abnormalities of CRBN or substrates, CRBN-independent mechanisms of IMiD resistance must exist. Drug resistance emerges universally with currently-used MM therapies, with intrinsic clonal heterogeneity of MM and selection of resistant clones playing a major role (78). The mechanisms by which resistant clones escape the effect of IMiDs may involve aberrant persistence of oncogenic signals, rewiring of transcription regulation to bypass dependence on IMiD-CRBN targets, or immune escape mechanisms that are not yet understood (57, 63). Ikaros-independent activation of the IRF4-MYC axis to promote MM cell proliferation *via* several routes has been described. Firstly, lenalidomide-resistant MM cells lacking CRBN abnormalities show upregulated IL-6/STAT3 signaling, leading to IRF4 persistence (79). Secondly, gene expression profiling of IMiD-resistant MM cell lines and small numbers of IMiD-resistant MM patients showed dysregulation of Wnt/β-catenin activity, leading to upregulated cyclin D1 and MYC (80). CD44, a known mediator of cell adhesion-mediated drug resistance, is also a downstream β-catenin target and may mediate IMiD resistance through enhancing MM cell adhesion to stromal cells (81). Inhibition of CD44 using ATRA was able to re-sensitize resistant MM cell lines to lenalidomide (80). These pathways, and others, could sustain persistent IRF4 and MYC oncogenic signaling to promote MM cell survival despite functional IMiD-mediated target degradation. The transmembrane proteins which CRBN co-chaperones (CD147-MCT1, LAT1/CD98hc) were found to resist IMiD-induced destabilization in IMiD-resistant MM cell lines, by an unknown mechanism (20, 21).

Ng et al. recently combined proteomic and RNA-sequencing approaches on longitudinal samples from relapsing MM patients to identify several CRBN-independent proteins that were aberrantly expressed (82). Of these, cyclin-dependent kinase 6 (CDK6) specifically impaired sensitivity of MM cell lines to IMiDs. They identified a network of CDK6-specific protein targets that were strongly associated with relapse, and proposed CDK6 as a master regulator of a relapsed-protein signature and driver of IMiD resistance. Future proteomic studies may provide further insight into additional CRBN-independent proteins that contribute to IMiD resistance, and assessment of the causality of these mechanisms in clinical IMiD resistance is necessary. Finally, it should be noted that prognostic markers of ‘high-risk’ myeloma (e.g. translocations t(4;14), t(14;16), del17p, gain/amp1q21 or gene expression signatures SKY92 and UAMS GEP70), are associated with early relapses after IMiD-based induction regimes (83). This covers a wide range of biology, and the drug resistance that emerges more rapidly in ‘high-risk’ patients is not specific to IMiDs. The interplay between the drivers of a ‘high-risk’, early-relapsing myeloma phenotype and specific mechanisms of IMiD resistance requires further study.

### Tumor microenvironment IMiD resistance mechanisms

Immune cell-mediated mechanisms of IMiD resistance are likely to be important but are not well studied or understood. Although IMiD drugs activate T cells, deep immune-profiling studies on IMiD-resistant patients have demonstrated an expansion of exhausted effector T cell populations with increased lymphocyte activating 3 (LAG3) and T cell immunoreceptor with Ig and ITIM domains (TIGIT) checkpoint inhibitor expression (84). Increased LAG3 expression as a marker of T cell exhaustion correlated with a worse PFS. This is consistent with other evidence that T cell exhaustion is a feature of MM relapse (31, 85). Addition of daratumumab to IMiDs has successfully overcome IMiD resistance *via* synergistic immune cell activation (86), and more potent CELMoDs may revitalize anti-tumor immunity in heavily pre-treated patients with prior IMiD resistance and features of T cell exhaustion (87). In this study, large-scale mass cytometry profiling of bone marrow tumor microenvironment of iberdomide patients demonstrated significant expansion and activation of T and NK cells, which was postulated to help overcome prior IMiD resistance. Evidence therefore suggests a role for T cell exhaustion and loss of anti-MM immunity in IMiD resistance. However, the degree of importance of immune-mediated versus MM cell-specific mechanisms of resistance is unclear, and interplay between IMiD-resistant MM clones and exhausted immune cell populations requires ongoing study.

## Conclusion

IMiDs have revolutionized outcomes for patients, but they do not cure myeloma, and drug resistance consistently emerges. To target IMiD-resistant MM with enhanced therapeutic combinations, and so prolong patient survival, is a tantalizing goal. With the revolutions in myeloma care that novel immunotherapies are bringing, the optimal placement of IMiDs and their CELMoD derivatives in therapeutic sequencing and combinations will be an ongoing subject of research and debate. In order to achieve best outcomes in this era of ever-growing myeloma treatment choices, genomic and/or other biomarkers of drug efficacy or resistance will be needed to guide clinical decision making for individual patients. Understanding some of the genetic causes of IMID resistance, such as CRBN disruption, will enable their incorporation into targeted sequencing assays that are increasingly deployed in clinical practice. However, the contribution to IMiD resistance of immune cell suppression and exhaustion is less clear, and more work is needed to understand whether assays of immune cell number or function could contribute clinically-relevant guidance on how and when to use this class of drugs. Nonetheless, the scope for better precision in deployment of cereblon targeting agents will no doubt be part of the evolving approach to improving outcomes in myeloma.

## Author contributions

LC reviewed the literature and wrote the initial draft. SG edited and co-wrote the final draft. All authors contributed to the article and approved the submitted version.

## Funding

LC is funded by the International Myeloma Society Career Development Award. SG holds research funding from Cancer Research UK (RCCCSF-Nov21\100004), Innovate UK (Project no. 50234) and works in a Medical Research Council (MRC)-funded institute.

## Acknowledgments

We thank our funders, and Prof Anjan Thakurta for helpful discussions on the topic. Figures created with BioRender.com.

## Conflict of interest

SG receives research funding from Bristol Myers Squibb.

The remaining author declare that the research was conducted in the absence of any commercial or financial relationships that could be construed as a potential conflict of interest.

## Publisher’s note

All claims expressed in this article are solely those of the authors and do not necessarily represent those of their affiliated organizations, or those of the publisher, the editors and the reviewers. Any product that may be evaluated in this article, or claim that may be made by its manufacturer, is not guaranteed or endorsed by the publisher.
